# Factors associated with meeting physical activity guidelines during the COVID-19 pandemic

**DOI:** 10.1186/s12889-022-14613-8

**Published:** 2022-11-25

**Authors:** Natalia I. Heredia, Michael Machiorlatti, Belinda M. Reininger, Candace Robledo

**Affiliations:** 1 School of Public Health, The University of Texas Health Sciences Center at Houston, Houston, TX USA; 2 School of Medicine, Department of Population Health and Biostatistics, The University of Texas Rio Grande Valley, Edinburg, TX USA; 3 School of Public Health, The University of Texas Health Science Center at Houston, Brownsville Regional Campus, Brownsville, TX USA

**Keywords:** Physical activity, Neighborhood safety, Pet ownership, Income, COVID-19 pandemic

## Abstract

**Introduction:**

The COVID-19 pandemic impacted individual physical activity levels. Less is known regarding how factors such as sociodemographic and built environment were associated with physical activity engagement during the pandemic. Understanding these factors is critical to informing future infectious disease mitigation policies that promote, rather than hinder physical activity. The purpose of this study was to assess predictors of physical activity levels during the beginning of the pandemic (April-June 2020), including Stay-at-Home length and orders, neighborhood safety, and sociodemographic characteristics.

**Methods:**

Data included 517 participants who responded to an anonymous online survey. Physical activity was assessed with a modified Godin Leisure-time exercise questionnaire. We used logistic regression models to estimate unadjusted and adjusted odds ratios (aOR) and their 95% confidence intervals (CI) for the associations between independent variables (e.g., demographic variables, neighborhood safety, COVID Stay-at-Home order and length of time) and physical activity levels that did not meet (i.e., < 600 metabolic equivalents of task [MET]-minutes/week) or met guidelines (i.e., ≥ 600 MET-minutes/week). We used R-Studio open-source edition to clean and code data and SAS V9.4 for analyses.

**Results:**

Most participants were 18–45 years old (58%), female (79%), Hispanic (58%), and college/post-graduates (76%). Most (70%) reported meeting physical activity guidelines. In multivariate-adjusted analyses stratified by income, in the highest income bracket (≥ $70,000) pet ownership was associated with higher odds of meeting physical activity guidelines (aOR = 2.37, 95% CI: 1.23, 4.55), but this association did not persist for other income groups. We also found lower  perceived neighborhood safety was associated with significantly lower odds of meeting physical activity guidelines (aOR = 0.15, 95% CI:0.04–0.61), but only among individuals in the lowest income bracket (< $40,000). Within this lowest income bracket, we also found that a lower level of education was associated with reduced odds of meeting physical activity guidelines.

**Discussion:**

We found that perceived neighborhood safety, education and pet ownership were associated with meeting physical activity guidelines during the early months of the COVID-19 pandemic, but associations differed by income. These findings can inform targeted approaches to promoting physical activity during subsequent waves of COVID-19 or future pandemics.

## Introduction

The COVID-19 pandemic caused global changes in the way people conduct their daily lives. For instance, shelter in place/stay at home mandates (heretofore Stay-at-Home) enacted to help reduce community transmission restricted the movement of individuals [1]. Restricted movement, in turn, may have contributed to the reduction in physical activity across many demographic groups [2–7]. However, the reasons for the decreases in physical activity during COVID-19, or more generally, the factors associated with physical activity during this time, are still not well understood. Understanding these factors is critical to informing future infectious disease mitigation policies that promote, rather than hinder physical activity [8]. This is especially important given evidence of the positive impact of physical activity on mental health during the pandemic [9], immune response to vaccination [10], COVID-19 outcomes [11], and critically, that physical inactivity will continue to be an important risk factor in future pandemics [12–14].

Less is known about factors associated with physical activity during the COVID-19 pandemic among U.S. adults. In the U.S., COVID-related state and municipal ordinances included the wide-spread closure of gyms and open spaces, such as parks, for prolonged periods of time [15–17], and the closing of some public open spaces, such as parks [18, 19]. If indoor recreational facilities were closed, but outdoor green spaces remained open, individuals living in areas with fewer of these public outdoor spaces or in unsafe neighborhoods may have experienced reduced opportunities for physical activity [20–22]. Prior to the pandemic, studies had demonstrated that the neighborhood environment, including perceived safety, were associated with physical activity [23], but it is unknown if and how these factors would impact physical activity during the COVID 19 pandemic. However, under Stay-at-Home order, higher income individuals who tend to have more access to yard space or exercise equipment in the home [24–26] may not have been as affected as low-income individuals. Considerable gaps remain in understanding how Stay-at-Home orders and neighborhood safety impacted physical activity during the COVID-19 pandemic.

Similarly, knowledge regarding how sociodemographic characteristics are associated with physical activity during the pandemic could help inform future promotional strategies for specific individuals and/or communities. For instance, while pet ownership has been found to be associated with physical activity in other countries during COVID-19 [27], this relationship has yet to be investigated with a U.S. sample during the COVID-19 pandemic. In this same context, even with restricted movement, pet owners may still need to go outside the home to tend to their pets’ needs.. Moreover, the physical activity of adults may have been negatively impacted in households with school-age children due to remote learning [28, 29]. Lastly, while socioeconomic factors are known to be associated with physical activity, including income and education [30], it is important to confirm this relationship held during the COVID-19 pandemic.

The purpose of this study is to assess the association of Stay-at-Home orders and length, neighborhood safety, and demographics variables with meeting physical activity guidelines in a predominately Hispanic and female group of survey respondents during the initial phase of the COVID-19 pandemic (April to June, 2020).

## Methods

### Data collection and study design

The current study is a cross-sectional analysis using data from the COVID-19 Impact on Health and Well-being Survey (IHWS). COVID-19 IWHS was designed to examine how COVID-19 pandemic Stay-at-Home order impacted mental health and well-being [31]. Members of the Departments of Population Health & Biostatistics, Family Medicine, Psychological Sciences and Sociology at University of Texas Rio Grande Valley (UTRGV) developed this survey. The survey collected sociodemographic information along with information on pandemic-related factors. Several validated instruments such as the General Anxiety Disorder(GAD)-7, Patient Health Questionnaire (PHQ)-9, and Perceived Stress Scale were used in this survey. All instruments were translated into Spanish when a validated Spanish translation did not exist. The instruments were administered in English and Spanish using the Research Electronic Data Capture (REDCap) platform, a secure web application for building and managing online surveys and databases.

We used snowball sampling methods to recruit English- or Spanish-speaking adults via e-mail and social media from April 20, 2020 to July 1, 2020. Participants responded to the anonymous online survey and did not receive an incentive for their participation. Surveys were accessed by clicking a public-facing survey link or a QR code from a flyer or social media banner/graphic. Informed consent was obtained from all participants; participants were presented with a consent statement, with language included to indicate they were granting their consent to participate by completing the survey, allowing us to maintain anonymity. This study was approved by the Institutional Review Board at UTRGV.

### Study population

**Fig. 1:**
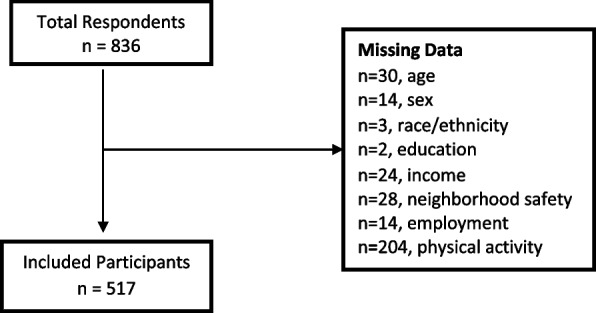
Flow Diagram for Inclusion of Participants

A total of 836 individuals completed the survey. About 82% of respondents were from Texas, with the majority of those respondents located in two major counties in the Lower Rio Grande Valley: Cameron (*n* = 105, 20.3%) and Hidalgo (*n* = 186, 36.0%) counties. The remaining 18% of the overall sample were scatter across 28 other U.S. states spanning all of the major regions in the U.S. We excluded participants with missing data on demographics, neighborhood safety, and physical activity (See Fig. 1), resulting in a final sample of 517.

### Measures

Data was anonymously collected via REDCap v 9.1.1 in both Spanish and English. Among other variables, the questionnaire collected demographics, physical activity, and neighborhood safety.

#### Demographics

We collected information on age (18–45, 45–64, 65 +), sex (male, female), race/ethnicity (Hispanic, Non-Hispanic White, Non-Hispanic Other), education level (“High School or less” i.e. did not finish high school, finished high school, or completed GED; “Some College” i.e. some college, associate’s degree, and/or technical school; “College and beyond” i.e. college, post-graduate or professional school), annual household income (< $40,000, $40,000 -$69,999, $70,000 or more), employment status (employed, unemployed), pet ownership (yes, no), and number of children (0, 1, 2, 3 +).

#### Physical activity

We used a modified Godin Leisure-Time Exercise Questionnaire to assess physical activity [32]. Example questions from the Godin scale included: “During a typical 7-day period (a week), how many times on average do you do moderate exercise, not exhausting, (e.g. fast walking, baseball, tennis, easy bicycling, volleyball, easy swimming, dancing) for more than 10 min during your free time?” This Godin scale is modified to include duration (e.g., “On occasions when you do moderate exercise, what is the average number of minutes you exercise?”). From these responses, we calculated metabolic equivalent of task (MET)-minutes using moderate and strenuous activity variables to get total MET-minutes of physical activity per week, and finally dichotomized into meeting or not meeting physical activity guidelines with the cutoff of ≥ 600 MET-minutes per week, per the Physical Activity Guidelines for Americans [33].

#### Neighborhood safety

Neighborhood Safety was collected with a 9-item measure. Participants were asked to rate how much all of the following were problems in their neighborhood: loud noise, litter, people using/selling drugs, crime, no safe place for children to play, not safe to walk alone at night, stray dogs and other animals. Participants were told to consider their neighborhood to be the area within a 5-minute walking distance from their home. The response scale for the first 7 items was “not a problem”, “some problem”, and “a big problem”. For the final two items, participants were also asked to compare their neighborhood to others in the region and what they think of their neighborhood as a place to live. The range for the scale was 9 to 26, with higher numbers indicating ***less*** neighborhood safety. Cronbach’s alpha was 0.84, indicating good internal consistency. We also divided the variable into tertiles to examine the relationship with physical activity, with Tertile 3 representing the lowest levels of perceived neighborhood safety. 

#### COVID-19 stay-at-home orders

We collected two variables related to COVID-19. The first asked if individuals were currently living under a Shelter-in-Place or Stay-at-Home order, with “yes”, “no” or “I don’t know” as response options. The second question asked individuals when the first day was they were asked to stay home, from which we calculated the length of the Stay-at-Home order.

### Statistical analyses

Descriptive statistics (Table 1) were created for all covariates. We used binary logistic regression to produce unadjusted odds ratios (OR) on the association of the predictors with physical activity. For our final adjusted model, we included age, race and sex along with covariates with p -values < 0.25 in unadjusted associations. Two-way interactions were explored in the set of variables screened into the final model. An interaction was deemed to be significant if *p*-value < 0.05 and stratified analysis was then performed. All covariates screened in remained in the final adjusted model and *p*-value < 0.05 was used to determine significance in the final model. SAS 9.4 was used to perform all analyses.

**Table 1:** Characteristics of the Sample (*n*=517)

Variable	Class	n (%)
Age	18–45	299 (57.8)
	45–64	170 (32.9)
	65 +	48 (9.3)
Sex	Male	110 (21.3)
	Female	407 (78.7)
Race/Ethnicity	Hispanic	297 (57.5)
	Non-Hispanic White	191 (36.9)
	Non-Hispanic Other	29 (5.6)
Education Level	High School or Less	27 (5.2)
	Some College	96 (18.6)
	College and beyond	394 (76.2)
Annual Household Income	< $40,000	126 (24.4)
	$40,000 to $69,999	110 (21.3)
	≥ $70,000	281 (54.4)
Employment Status	Employed	402 (77.8)
	Unemployed (all reasons)	115 (22.2)
Children	0	231 (50.3)
	1	85 (18.5)
	2	90 (19.6)
	3 +	53 (11.6)
Pet Ownership	No	148 (29.0)
	Yes	362 (71.0)
Currently under Stay-At-Home Order	No	110 (22.2)
	Yes	386 (77.8)
Length of Stay-At-Home Order	< 30 Days	441 (85.3)
	≥ 30 Days	76 (14.7)
Neighborhood Safety, M (SD)	12.0 (3.3)	-
Neighborhood Safety Tertiles	9–10	221 (42.8)
	11–12	137 (26.5)
	13 or more	159 (30.8)
Total MET-minutes, M(SD)	1983.9 (1753.6)	-
Meeting Physical Activity Guidelines	No	153 (29.6)
	Yes	364 (70.4)
Vigorous Activity Change	Less	230 (45.1)
	Same	207 (40.6)
	More	73 (14.3)
Moderate Activity Change	Less	193 (37.8)
	Same	212 (41.6)
	More	105 (20.6)
Mild Activity Change	Less	178 (35.1)
	Same	250 (49.3)
	More	79 (15.6)

## Results

The average age of respondents was 42.7 years old (Table 1). The majority of respondents were female, Hispanic, had a bachelor’s degree or higher, made more than $70,000 per year, were employed, had a pet and no children. Most respondents reported there was a Stay-at-Home order where they lived at the time they completed the questionnaire (78%), which is not surprising given 56% of the sample came from two neighboring counties on the Texas-Mexico border. At the time the survey was administered, the average amount of time respondents reported being under a Stay-at-Home order was 49.5 days, with a minimum of 31 days and a maximum of 110 days (data not shown). As a result of the pandemic, 46% of participants said they increased all types of intensities of activity (mild, moderate and vigorous) with all others reporting some variation in their activity levels.

**Fig. 2:**
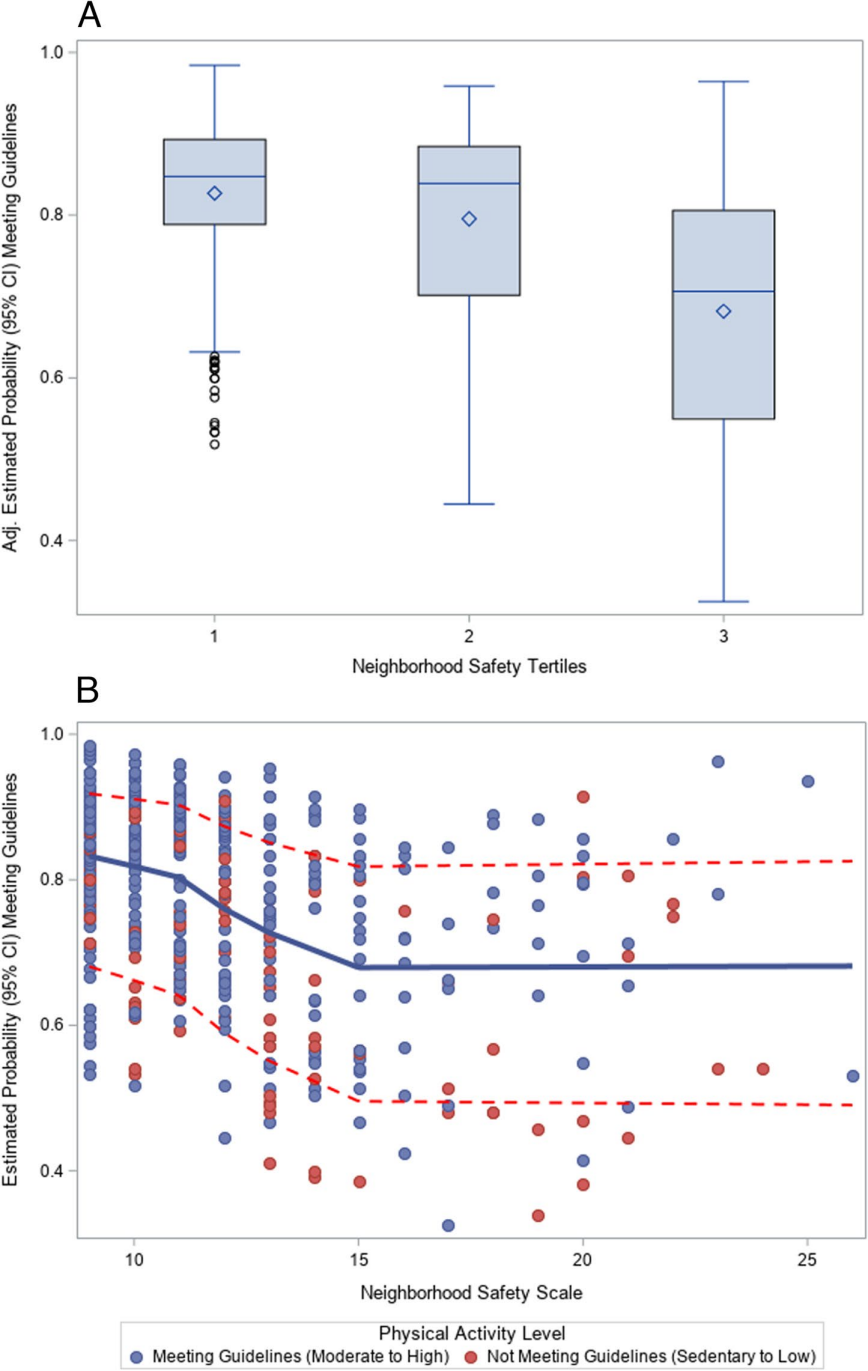
**A** Adjusted Prediction of Meeting Physical Activity Guidelines by Tertiles of Neighborhood Safety Scale. Adjusted for age, race, sex, education level, income level pet ownership, length of stay at-home order and change in physical activity level. Neighborhood Safety Tertiles represent the highest (Tertile 1) to lowest (Tertile 3) levels of perceived neighborhood safety. **B** Adjusted Prediction of Meeting Physical Activity Guidelines by Continuous Neighborhood Safety Scale

We explored the association between neighborhood safety and physical activity. In the adjusted association of neighborhood safety tertiles with meeting physical activity guidelines, we see, on average, each subsequent tertile (progressing from most to least safe) is associated with lower odds of meeting physical activity guidelines (Fig. 2A). In the adjusted association of the continuous neighborhood safety scale with meeting physical activity guidelines, we see an inverse association between the two variables, which then levels off at values higher than 15, indicating that higher levels on the neighborhood safety scale (i.e. the highest perception of safety) no longer impact the probably of meeting physical activity guidelines (Fig. 2B).

We found significant associations between race/ethnicity, education, length of Stay-at-Home order, and neighborhood safety tertiles with physical activity level before adjustment (Table 2). We tested the interaction of both income and sex separately with physical activity, and found a statistically significant interaction with income. Therefore, we stratified all results by income. Among individuals in the lowest income group (i.e. < $40,000), those with some college as compared to a college degree or higher had 74% lower odds of meeting physical activity guidelines. Among individuals in the < $40,000 income group, living in the most unsafe neighborhood (i.e. tertile 3) was associated with an 85% lower odds of meeting physical activity guidelines as compared to those in the 1^st^ tertile (safest neighborhood). We found no statistically significant association between neighborhood safety and physical activity in either the $40,000—$69,999 or ≥ $70,000 income groups. Lastly, we found a strong, statistically significant association between pet ownership and physical activity, but only in the highest income group (i.e. ≥ $70,000).

**Table 2:** Unadjusted and Adjusted Logistic Regression Results of the Association of Each Covariate with Meeting Physical Activity Guidelines

Variable	Class	OR (95% CI)	AOR (95% CI)		
			**Income Group**		
			< $40,000	$40,000 to $69,999	≥ $70,000
Age Category	18–44 (REF)				
	45–64	0.91 (0.58, 1.42)	0.88 (0.23, 3.40)	0.69 (0.26, 1.81)	0.87 (0.44, 1.71)
	65 +	1.25 (0.58, 2.71)	0.84 (0.11, 6.72)	NE	0.91 (0.30, 2.72)
Sex	Female vs Male	1.01 (0.61, 1.67)	0.77 (0.28,2.15)	1.36 (0.41, 4.57)	1.01 (0.47, 2.18)
Race/Ethnicity	Non-Hispanic White (REF)				
	Hispanic	0.55 (0.35, 0.86) **	2.04 (0.47, 8.89)	1.16 (0.36, 3.70)	0.62 (0.31, 1.24)
	Non-Hispanic Other	2.82 (0.64, 12.44)	NE	NE	2.35 (0.48, 11.54)
Educational Level	High School or less	0.60 (0.25, 1.41)	0.72 (0.21, 2.44)	0.93 (0.08, 10.24)	NE
	Some College	0.55 (0.34, 0.91) *	0.26 (0.09, 0.71) **	1.44 (0.45, 4.56)	1.10 (0.42, 2.90)
	College and beyond (REF)				
Employment Status	Unemployed vs Employed	0.83 (0.51, 1.35)	-	-	-
Children	0 (REF)				
	1	1.51 (0.82, 2.81)	-	-	-
	2	1.23 (0.69, 2.19)	-	-	-
	3 +	1.72 (0.79, 3.72)	-	-	-
Pet Ownership	Yes vs No	1.37 (0.88, 2.14)	0.53 (0.19, 1.49)	1.41 (0.50, 3.98)	2.37 (1.23, 4.55) **
Currently Under Stay-At-Home Order	Yes vs No	1.33 (0.81, 2.17)	-	-	-
Length of Stay -At-Home Order	≥ 30 days vs < 30 Days	2.41 (1.17, 5.00) *	3.74 (0.45, 31.23)	1.59 (0.154, 16.29)	1.95 (0.73, 5.21)
Neighborhood Safety Tertiles	1 (Ref)				
	2	0.81 (0.47, 1.39)	0.43 (0.09, 1.99)	0.90 (0.25, 3.22)	0.83 (0.40, 1.75)
	3	0.44 (0.27, 0.71) **	0.15 (0.04, 0.61) **	0.53 (0.18, 1.58)	0.66 (0.28, 1.55)

C-statistic for AOR models are: 0.758; 0.644; 0.660; *p-value < 0.05, **p-value < 0.01

*NH* non-Hispanic, *AOR* Adjusted Odds Ratio; AOR was adjusted for age, sex, race, education, income, pet, length of stay-at-home and NHS. *NE* No Estimate Due to Low Sample Size in Subgroup

## Discussion

In this assessment of U.S. adults, representing a predominately Hispanic, Texas-based sample, we found lower levels of perceived neighborhood safety (as compared to the highest level of perceived neighborhood safety) were associated with lower odds of meeting physical activity guidelines, but only in adults reporting a household income of less than $40,000 a year. These findings mirror those found by colleagues, who identified that the COVID-19 pandemic decreased use of outdoor spaces in low socioeconomic status (SES) areas, but actually increased use in high SES areas [25]. Our findings could be because of the confluence of unsafe neighborhoods with low income or low SES neighborhoods [34, 35], or could be because above a certain income, individuals have sufficient disposable income to pay for equipment in their homes or have enough private outdoor area on their property to allow them to maintain their physical activity [24–26]. Importantly, irrespective of income bracket, we saw an inverse association between neighborhood safety and physical activity, but this leveled off at values on the neighborhood safety scale higher than 15 (i.e. the least safe neighborhoods). This leveling off at 15 would seem to indicate that higher levels on the neighborhood safety scale (i.e. the highest perception of safety) no longer impact the probability of meeting physical activity guidelines. Individuals only need a specific threshold of safety met for them to feel comfortable in outdoor spaces.

As compared to those with a college education or higher, holding an Associate's degree, completing technical school, or some college was associated with lower odds of meeting physical activity guidelines, but only in adults reporting a household income of less than $40,000 a year. Previous data has shown an association of low income and lower education with lower levels of physical activity [36, 37]. These findings may reflect the overall patterns of health inequities or the demand on many essential workers during the pandemic [38–41], many of whom have jobs requiring less education and tend to receive lower salaries. It also may reflect that those with lower incomes had to continue working in-person despite the COVID-19 risks and therefore had less time to be physically active. Our study contributes to the literature on the association of lower education and lower income with lower levels of physical activity during the early part of the pandemic.

Moreover, we found pet ownership in the highest income bracket (≥ $70,000) was associated with increased odds of meeting physical activity guidelines, though this statistically significant association was not found in the two lower income brackets. A similar association was found in a study conducted in Singapore in an affluent sample [27]. Stay-at-Home orders and other business-specific decisions to continue working at home during the first four months of the pandemic favored office workers and those with higher income [41, 42], and thus, may have provided more time for these at-home workers with pets, particularly those with dogs, to get outside and engage in more physical activity. It is important to note that regardless of the COVID-19 pandemic, dog-ownership specifically is associated with walking [43, 44], so these findings may reflect a persistent underlying association.

There are several limitations to this study. Foremost, this was a cross-sectional study, which limits our ability to infer causation and observe how these same individuals were impacted by social and demographic factors before and well after the first few months of the COVID-19 pandemic. Our sample was a convenience sample of individuals willing to complete an online survey, thus, our results are not representative of any specific demographic group and it should be assumed that individuals with less access to reliable internet may have been more likely to be excluded from this sample. More specifically, we had a limited number of men included in this study (21%). We also did not assess the actual neighborhood environment, so it is possible that those reporting “unsafe” neighborhoods may not truly live in one. However, research shows perceived, rather than objective, neighborhood environment is more critical for physical activity [23].

## Conclusion

We found perceived neighborhood safety, educational levels and pet ownership were associated with meeting physical activity guidelines during the early months of the COVID-19 pandemic, though associations varied by annual household income. These findings can inform approaches to promoting physical activity during subsequent waves of COVID-19 or future pandemics, potentially targeting different SES groups with messaging most appropriate to their needs.

## Data Availability

The dataset supporting the conclusions of this article is available upon request from Michael Machiorlatti at michael.machiorlatti@utrgv.edu.
